# Perceived coercion in psychiatric inpatients: a validation study of the Romanian-language version of the Admission Experience Survey

**DOI:** 10.1080/13218719.2024.2372767

**Published:** 2024-09-08

**Authors:** Radu-Mihai Păun, Neculai Alexandru Pavel, Valentin-Petre Matei, Cătălina Tudose

**Affiliations:** a Department of Psychiatry, ‘Carol Davila’ University of Medicine and Pharmacy, Bucharest, Romania; b ‘Prof. Dr. Alexandru Obregia’ Clinical Psychiatric Hospital, Bucharest, Romania

**Keywords:** aggression, coercion, involuntary admission, psychiatric emergencies, questionnaire, severe mental illness

## Abstract

The MacArthur Admission Experience Survey was developed to evaluate perceived coercion in psychiatric inpatients and comprises of three subscales: “Perceived coercion”, “Negative pressures” and “Voice”. The present study aims to evaluate the psychometric properties of a Romanian language version of the AES (R-AES) and to identify predictors for higher perceived coercion. We developed the R-AES using a translation/back-translation procedure and administered it to 137 patients admitted to the “Profesor Doctor Alexandru Obregia” psychiatric hospital in Bucharest, Romania. Discriminatory power was evaluated by comparing the scores of voluntarily and involuntarily admitted patients. The R-AES showed satisfactory internal consistency (Crohnbach’s alpha = 0.92). Principal components analysis disclosed a three-factor solution explaining 69.1% of variance. Factor components roughly corresponded to the original subscales. Higher scores were associated with involuntary admission, objective coercive measures, younger age, frequent past hospitalisations, treatment non-compliance, psychosis, mania, greater disease severity, aggression, and presentation by police or ambulance.

## Introduction

Coercive measures, defined as measures carried out against the patient’s autonomous opposition or against his/her wishes (1), continue to be used often in the management of psychiatric emergencies (2). These include formal/objective measures, such as involuntary admission, restraint, seclusion, or forced treatment, as well as informal/subjective measures, such as persuasion, interpersonal leverage, inducement, and threats (3). While most countries relegate the use of formal coercive measures in psychiatry to persons who present an obvious danger to themselves or others (4), informal coercion is difficult to adequately define and regulate and thus remains widely used (3). It is argued that coercive practices infringe on patients’ rights, such as autonomy and freedom of movement, but it is generally accepted that a degree of coercion is justifiable when the patient’s safety and wellbeing are threatened (3).

Subjectively perceived coercion, understood as the person’s lived experience of being coerced, is reported by most involuntarily admitted patients and a significant number of voluntary patients (2,5,6). In addition to exposure to objective coercive measures, female sex (7), older age (8), poorer global functioning (9), psychotic symptoms (10), lack of insight (6), and lack of transparency in the legal and therapeutic decision process (10,11) have been associated with greater levels of perceived coercion. High perceived coercion is, in turn, detrimental to patient–clinician rapport (12) and may lead to poorer treatment adherence and worse long-term outcomes (13).

The Admission Experience Survey (AES) is a 16-item questionnaire developed by the MacArthur Research Network on Mental Health and the Law to measure the level of perceived coercion during mental hospital admission (14). It has consistently proven a useful and reliable assessment tool and has been validated for use in Chinese (15), Italian (16), French (17) and Swedish (18). Response items are grouped into three categories: perceived coercion, negative pressures, and voice. The perceived coercion subscale evaluates the patient’s feeling of control over his presentation and admission to the hospital, the negative pressures subscale evaluates the extent to which the patient felt directly or indirectly threatened into accepting hospitalization by the various actors involved, and the voice subscale refers to the degree to which the patient felt free to express his opinions and preferences during the admission process and whether or not they were taken into consideration.

At the present time there is no dedicated Romanian-language tool for evaluating coercion experienced by psychiatric inpatients. Therefore, the aim of our study is to assess the psychometric properties of a Romanian version of the AES (R-AES) (See Supplementary material).

## Method

### Participants

A total of 112 patients admitted in the ‘Profesor Doctor Alexandru Obregia’ psychiatric hospital in Bucharest between September 2022 and January 2023 were recruited to take part in the study. Sociodemographic and clinical data were collected by interview and from patient charts and were corroborated by the attending physician and family when needed. Patients not fluent in Romanian and/or suffering from a major neurocognitive disorder according to DSM-5 criteria were excluded. The study was conducted in accordance with the Helsinki Declaration (the World Medical Association, Inc. Declaration of Helsinki Ethical Principles for Medical Research Involving Human Subjects) and approved by the local ethics board. All patients gave written informed consent for participation.

Mean age was 47.3 years, and 44.6% (*n* = 50) participants were female. Involuntary admissions accounted for 40.2% (*n* = 45) of cases. Primary diagnoses were established at admission by the physician conducting the hospitalisation, according to ICD-10 criteria. We grouped these in the following categories: schizophrenia and other psychotic disorders (ICD-10 codes F20–F29), mania 13.4% (ICD-10 codes F31.0–F31.2), depression (ICD-10 codes F31.3–F34), alcohol and substance use disorders (ICD-10 codes F10–F19), and others. Disease severity was assessed using the Clinical Global Impression Severity Scale (CGI-S) (19), which assigns a numerical score from 1 to 7 based on the rater’s clinical experience, with a score of 1 describing a patient who is ‘not at all ill’ and 7 ‘among the most extremely ill patients’. Aggression was evaluated using the modified Overt Aggression Scale (mOAS) (20) which assesses four domains: verbal aggression, property aggression, auto-aggression, and physical aggression; higher scores (up to a maximum of 40) indicate more severe aggressive behaviours. Treatment adherence, which we defined as taking 80–120% of prescribed psychiatric treatment dose in the 4 weeks before the current admission (or between the previous hospital discharge and present admission if they were less than 4 weeks apart), was evaluated only when at least two outside sources were available for corroboration (medical files, family, friends, attending physician, general practitioner).

Objective coercive measures (OCM), defined as isolation, forced treatment, or physical restraint, were used in 10.94% (21) of cases and were documented using patient files.

#### Validation

The original AES included 15 binary response items grouped into three subscales: (a) Perceived coercion, (b) Negative pressures and (c) Voice, as well as a 16th item that assessed the patient’s feeling towards hospitalization. The scale was translated into Romanian by PR then translated back into English by an independent professional linguist. The back-translated version was compared to the original, and discrepancies were resolved via group consensus.

Using the known group method, the theoretical basis was that patients who had been admitted involuntarily would perceive higher coercion compared to voluntarily admitted patients, as evaluated by R-AES.

#### Statistics

All analyses were conducted using SPSS Statistics v26. The alpha value was set to 0.05, two tailed. Normality of data was assessed using the Shapiro–Wilk test. Since the data was not normally distributed, we used non-parametric statistical analyses. Item-to-total correlations were calculated using Spearman rank correlation coefficients (Table 1). The Mann-Whitney *U* test was to explore the association between R-AES scores and categorical variables, and afterwards Cohen’s *d* was added as a measure of effect size. The association between R-AES scores and continuous variables was explored by linear regression.

**Table 1.** Item-to-total Spearman rank correlation coefficients.

	Spearman ρ
Item 1	.81
Item 2	.72
Item 3	.55
Item 4	.84
Item 5	.73
Item 6	.53
Item 7	.77
Item 8	.66
Item 9	.70
Item 10	.64
Item 11	.62
Item 12	.73
Item 13	.75
Item 14	.86
Item 15	.76

Note. Higher values indicate higher correlation between the scored item and the total test score.

We performed factor analysis on the R-AES items in a two-step approach: first, using two factors as shown in Table 2, and, second, using three factors, as shown in Table 3. Oblique rotation with a delta value of 0 was used. We analysed the R-AES sub-groups’ internal consistency using Cronbach’s alpha.

**Table 2.** Factor analysis (2 factors) pattern matrix.

	Component	
Items	1	2
Factor 1		
14. I had a lot of control over whether I went to the hospital	.85	
9. No one seemed to want to know whether I wanted to come into the hospital	.83	
1. I felt free to do as I wanted about coming into the hospital	.82	
4. I chose to come into the hospital	.82	
3. I had enough of a chance to say whether I wanted to come into the hospital	.76	
15. I had more influence than anyone else on whether I came into the hospital	.68	
7. It was my idea to come into the hospital	.65	
5. I got to say what I wanted about coming into the hospital	.64	
13. My opinion about coming into the hospital did no matter	.45	
Factor 2		
10. I was threatened with commitment		.90
11. They said they would make me come into the hospital		.87
8. Someone physically tried to make me enter the hospital		.71
12. No one tried to force me to come into the hospital		.64
6. Someone threatened me to get to come into the hospital		.62
2. People tried to force me to come into the hospital		.59
Factor correlation	.55	
Cronbach’s alpha	.92	.87
Variance explained	52.6	10.5

**Table 3.** Factor analysis (3 factors) pattern matrix.

Items	Factor		
	1	2	3
Perceived coercion (Factor 1)			
9. No one seemed to want to know whether I wanted to come into the hospital	.92		
4. I chose to come into the hospital	.90		
14. I had a lot of control over whether I went to the hospital	.83		
7. It was my idea to come into the hospital	.78		
1. I felt free to do as I wanted about coming into the hospital	.66		
15. I had more influence than anyone else on whether I came into the hospital	.55		
External pressure (Factor 2)			
11. They said they would make me enter the hospital		.90	
10. I was threatened with commitment		.87	
8. Someone physically tried to make me come into the hospital		.87	
12. No one tried to force me to come into the hospital		.67	
6. Someone threatened me to get to come into the hospital		.58	
2. People tried to force me to come into the hospital		.48	
13. My opinion about coming into the hospital did no matter		.41	
Choice expression (Factor 3)			
3. I had enough of a chance to say whether I wanted to come into the hospital			.85
5. I got to say what I wanted about coming into the hospital			.57
Factor correlation			
F1 vs F2	.57		
F1 vs F3	.43		
F2 vs F3	.28		
Cronbach’s alpha	.92	.88	.64
Variance explained	52.6	10.5	6.0

## Results

Distribution of the total score of the R-AES had a mean of 3.18, *SD* of 4.24, range of 0–14, skewness of 1.26, and kurtosis of 0.28. We excluded Item 9 from that analysis as previously described in other validation studies. The item-to-total correlations (Table 1) ranged from .35 to .76, indicating that the R-AES is not a homogenous scale. Internal consistency was satisfactory, with a Cronbach’s alpha value of .92.

In the 2-factor model, Factor 1 corresponded to perceived coercion, explaining − 52.6% of the variance, while Factor 2 corresponded to negative pressures perceived by the patient, explaining 10.5% of the variance (In the second model, using 3 factors, Factor 1 corresponded to perceived coercion, Factor 2 to external pressure and Factor 3 to choice expression explaining 52.6%, 10.5% and 6% of the total variance. Based on the two models we considered the 3-factor approach to be more suitable.

There were statistically significant differences between voluntary and involuntary patients both in total R-AES score and in individual Factor 1 (*p* < .001), Factor 2 (*p* < .001) and Factor 3 (*p* < .001) scores (Table 4). Regarding the relationship between perceived coercion as and socio-demographic variables and medical history, younger age (*p* = .019), higher number of previous hospitalisations (*p* = .40) and treatment non-compliance (*p* = .023) (Table 5). Looking at clinical variables, patients with psychotic symptoms scored moderately higher than those with depression (*p* = .003, Cohen’s *d* = 0.66), while patients with mania scored moderately higher than those with depression (*p* = .008, Cohen’s *d* = 0.70) and those in the ‘Other’ diagnostic category (*p* = .05, Cohen’s *d* = 0.71). Greater disease severity measured by CGI-S (*p* = .004) and higher aggression as measured by the mOAS (*p* < .001) had a statistically significant association with higher R-AES scores (Table 6). Finally, regarding hospital presentation, police and ambulance presentations were associated with greater R-AES scores when compared to being brought by friends or family or self-referral, with the effect size being highest for police presentations (Table 7).

**Table 4.** Factor 1, 2, 3 and total AES mean score comparison between voluntarily and involuntarily admitted patients.

	Voluntarily admitted(*N* = 67)	Involuntarily admitted(*N* = 45)	Mann-Whitney *U*		Cohen’s *d*
			*z*	***p***	
Factor 1	0.59 ± 1.24	3.28 ± 2.57	−5.65	<.001	1.33
Factor 2	0.43 ± 0.93	3.07 ± 2.53	−6.55	<.001	1.39
Factor 3	0.21 ± 0.47	0.79 ± 0.86	−4.32	<.001	0.84
Total AES	1.16 ± 2.11	7.00 ± 4.89	−5.68	<.001	1.55

Note. AES = Admission Experience Survey.

**Table 5.** Socio-demographic and medical history variables and AES score.

	*n*	*B* coefficient^a^	Cohen’s *d*	*p* ^b^	
Age (years)		−.051		**.019**	
Education (years)		.014		.882	
Sex					
Male	74		0.09	.573	
Female	63				
Etnicity					
Romanian	119		0.10	.532	
Other	16				
Relationship status					
Single	89		0.25	.132	
In a relationship	48				
Locative status					
Living alone	33		0.18	.272	
Living with others	98				
Disease duration (years)		−.011		.765	
Number of psychiatric hospitalisations in the past 12 months		.445		**.040**	
In contact with medical services in the past 12 months					
Yes	109		0.03		.841
No	17				
Compliant to medical treatment					
Yes	68		0.38		**.031**
No	51				

Note. AES = Admission Experience Survey. Bolded values denote a statistically significant association.

^a^Linear regression. ^b^Mann-Whitney *U* test.

**Table 6.** Clinical variables and R-AES.

Diagnosis	*n*	Cohen’s *d*	*B* coefficient^a^	*p* ^b^
Schizophrenia and psychotic disorders	46			
vs mania		0.13		.585
vs depression		0.66		**.003**
vs SUD		0.37		.129
vs others		0.44		.087
Mania	19			
vs depression		0.70		.**008**
vs SUD		0.55		.088
vs others		0.71		**.050**
Depression	39			
vs SUD		0.28		.252
vs others		0.02		.944
Substance use disorders	20			
vs others		0.21		.518
Others	13			
Exposure to OCM				
Yes	19	0.89		**<.001**
No	114			
CGI-S score			.608	**.021**
MoAS score			.318	**<.001**

Note. R-AES = Romanian Admission Experience Survey. SUD = Substance use disorder. OCM = Objective coercive measures. CGI-S = Clinical Global Impression Severity Scale. MoAS = Overt Aggression Scale. Bolded values denote a statistically significant association.

^a^Linear regression.

^b^Mann-Whitney *U* test.

**Table 7.** Hospital presentation and AES.

Presentation	*n*	Cohen’s *d*	*p*a
Police	25		
vs ambulance		0.64	**.025**
vs family/friends		1.33	**<.001**
vs self-referral		2.2	**<.001**
Ambulance	27		
vs family/friends		0.59	**.011**
vs self-referral		0.93	**<.001**
Family/friends	46		
vs self-referral		0.26	.201
Self-referral	37		

Note. AES = Admission Experience Survey. Bolded values denote a statistically significant association.

^a^Mann-Whitney *U* test.

## Discussion

To our knowledge, this is the first paper examining coercion in the Romanian mental health system. There is a very limited selection of standardized psychiatric assessment tools validated for use in Romanian, all of which are symptom and severity rating scales. It is our hope that by introducing an instrument that focuses on the individual’s experience of psychiatric hospitalization we can foster further research and constructive debate on this topic.

The reliability of the R-AES was satisfactory (Cronbach’s alpha = .92). Our version of the AES reliably differentiated involuntary and voluntary patients both as a total score and as a two-factor model. Furthermore, it was able to discern between involuntary patients who were subjected to objective coercive measures and those who were not.

The original scale disclosed a three-factor solution (‘Perceived Coercion’ comprising items 1, 4, 7, 14, 15, ‘Negative Pressures’ comprising items 2, 6, 8, 10, 11, 12, and ‘Voice’ comprising items 3, 5, 13), For the Romanian version of the AES, we explored both a two-factor and a three-factor solution, with the three-factor approach emerging as the more suitable option. Factor loading in the R-AES was for the most part consistent with the original, but there were several important differences. Factor 1, corresponding to ‘Perceived coercion’, encompassed items 1, 4, 7, 9, 14, 15, the only difference from the original being the inclusion of Item 9. Interestingly, the original version excluded this item due to its low loading, whereas in our version this item loaded highest in the three-factor solution and second-highest in the two-factor solution. The most likely explanation is that, due to linguistic differences, the translated statement is easier to understand than the English original. Factor 2, corresponding to ‘Negative pressures’, encompassed items 2, 6, 8, 10, 11, 12, 13, while Factor 3, corresponding to ‘Voice’, encompassed Items 3 and 5. The Italian (16) and Chinese (15) versions defined three factors, with slightly different item loadings, and the Swedish (18) version defined four separate factors.

Of the socio-demographic and medical-history-related variables analysed, younger age, a history of frequent hospitalisations, and treatment non-adherence were associated with higher R-AES score in our cohort. Non-adherence is related to lack of insight and impaired functionality (22,23), which are, in turn, associated with higher perceived coercion (6,9). However, past research has yielded mixed results, with no singular variable emerging as a consistent predictor of higher perceived coercion (24). This is likely due to multiple factors such as methodological heterogeneity, the inherent difficulty of measuring subjective coercion, and the differences in social, legal, and cultural frameworks.

Patients brought to the hospital by police or ambulance scored significantly higher than did those brought by friends/family or who self-presented, with those brought by the police feeling most coerced. While we found no studies directly analysing the link between the type of hospital presentation and subjective coercion, an analysis of the outcomes of psychiatric emergencies in Switzerland showed that, while police involvement often leads to the use of coercive medical measures, this is because police tend to be called upon in cases of patients with severe symptoms and/or aggressive behaviour (25). This is in accordance with our results, as disease severity and aggression were correlated with subjective coercion in our cohort.

When comparing R-AES scores between diagnostic categories, patients with psychosis scored higher than did those with depression, and patients with mania scored higher than those with depression and those in the ‘Other’ diagnostic category, which is consistent with existing data that suggests that manic or psychotic patients are more likely to feel coerced (9). Greater disease severity and aggression were also associated with higher R-AES scores in our cohort.

There are several limitations to our study. First, the selection process excluded patients who were unable to respond due to sensorial and/or cognitive deficits. Assessing the lived experience of this subgroup remains a major unmet need, both in psychiatry and in general medical practice. Second, due to the nature of the questionnaire and study population, a degree of response bias, particularly acquiescence bias, is inevitable. Third, while the sample is adequate in composition, it is only of moderate size compared to other similar studies.

## Conclusion

The R-AES demonstrated satisfactory psychometric properties in assessing perceived coercion in psychiatric patients. As the first instrument of this kind translated in Romanian, it may serve as a foundation for further research into the mental health system in an area of the world where data on this topic are scarce.

## Supplementary Material

Supplemental Material English AES

Supplemental Material Romanian AES
