# Supplementary material for: Perceived coercion in psychiatric inpatients: a validation study of the Romanian-language version of the Admission Experience Survey
Source: Psychiatr Psychol Law. 2024 Sep 8;33(1):22–31. doi: 10.1080/13218719.2024.2372767 (PMC12857718; doi:10.1080/13218719.2024.2372767)
Supplement: Supplemental Material English AES [file TPPL_A_2372767_SM6267.docx]

| 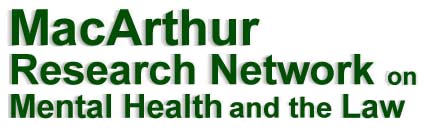  [Coercion: *Executive Summary*](https://web.archive.org/web/20170712054626/http:/www.macarthur.virginia.edu/coercion.html) \|[MacArthur Home](https://web.archive.org/web/20170712054626/http:/www.macarthur.virginia.edu/home.html)  **The MacArthur Coercion Study**  **Admission Experience Survey: Short Form**  "I am now going to read you some statements about your coming into the hospital this time. Please answer either "TRUE" or "FALSE" to each statement. Try to answer each question individually, no matter how similar it may sound to another."   \|  \| **True** \| **False** \| **Don't Know** \| \| --- \| --- \| --- \| --- \| \| **1.** I felt free to do what I wanted about coming into the hospital. \| **[ ]** \| **[ ]** \| **[ ]** \| \| **2.**People tried to force me to come into the hospital. \| **[ ]** \| **[ ]** \| **[ ]** \| \| **3.**I had enough of a chance to say whether I wanted to come into the hospital. \| **[ ]** \| **[ ]** \| **[ ]** \| \| **4.**I chose to come into the hospital. \| **[ ]** \| **[ ]** \| **[ ]** \| \| **5.** I got to say what I wanted about coming into the hospital. \| **[ ]** \| **[ ]** \| **[ ]** \| \| **6.**Someone threatened me to get me to come into the hospital. \| **[ ]** \| **[ ]** \| **[ ]** \| \| **7.** It was my idea to come into the hospital. \| **[ ]** \| **[ ]** \| **[ ]** \| \| **8.** Someone physically tried to make me come into the hospital. \| **[ ]** \| **[ ]** \| **[ ]** \| \| **9.** No one seemed to want to know whether I wanted to come into the hospital. \| **[ ]** \| **[ ]** \| **[ ]** \| \| **10.** I was threatened with commitment. \| **[ ]** \| **[ ]** \| **[ ]** \| \| **11.** They said they would make me come into the hospital. \| **[ ]** \| **[ ]** \| **[ ]** \| \| **12.** No one tried to force me to come into the hospital. \| **[ ]** \| **[ ]** \| **[ ]** \| \| **13.** My opinion about coming into the hospital didn't matter. \| **[ ]** \| **[ ]** \| **[ ]** \| \| **14.** I had a lot of control over whether I went into the hospital. \| **[ ]** \| **[ ]** \| **[ ]** \| \| **15.**I had more influence than anyone else on whether I came into the hospital. \| **[ ]** \| **[ ]** \| **[ ]** \| \| **16.** How did being admitted to the hospital make you feel?  Did it make you feel: \|  \|  \|  \| \| **a.** Angry. \| **[ ]** \| **[ ]** \| **[ ]** \| \| **b.**Sad. \| **[ ]** \| **[ ]** \| **[ ]** \| \| **c.** Pleased. \| **[ ]** \| **[ ]** \| **[ ]** \| \| **d.**Relieved. \| **[ ]** \| **[ ]** \| **[ ]** \| \| **e.**Confused. \| **[ ]** \| **[ ]** \| **[ ]** \| \| **f.**Frightened. \| **[ ]** \| **[ ]** \| **[ ]** \|   **Subscales of the MacArthur Admission Experience Survey (AES) -- Short Form 1**  *Item 9 of the AES-Short Form was eventually dropped from these scales.*  **I. Perceived Coercion Scale**  **The scale is thus 0-5, with each "True" = 0, and each "False" = 1. See Gardner et al (1993), p.316.**  **1.** I felt free to do what I wanted about coming into the hospital. **4.**I chose to come into the hospital. **7.**It was my idea to come into the hospital. **14.**I had a lot of control over whether I went into the hospital. **15.**I had more influence than anyone else on whether I came into the   hospital.  **II. Negative Pressures Scale**  **2.** People tried to force me to come into the hospital. **6.** Someone threatened me to get me to come into the hospital. **8.**Someone physically tried to make me come into the hospital. **10.**I was threatened with commitment. **11.** They said they would make me come into the hospital. **12.** No one tried to force me to come into the hospital.[*reverse scored*]  **III. Voice Scale**  **The "voice" scale is an early version of what became the "procedural justice" scale (see Lidz et al, 1995).**  **3.** I had enough of a chance to say whether I wanted to come into the   hospital. **5.** I got to say what I wanted about coming into the hospital. **13.** My opinion about coming into the hospital didn't matter.[*reverse   scored*]  **IV. Affective Reactions to Hospitalization**  **16.**How did being admitted to the hospital make you feel? Did it make you feel:  **a.**Angry **b.** Sad **c.** Pleased **d.** Relieved **e.** Confused **f.** Frightened  This instrument is taken from  Gardner, W., Hoge, S., Bennett, N., Roth, L., Lidz, C., Monahan, J., and Mulvey, E. (1993). Two scales for measuring patients' performance perceptions of coercion during hospital admission. *Behavioral Sciences and the Law, 20,* 307-321. |
| --- | --- | --- | --- | --- | --- | --- | --- | --- | --- | --- | --- | --- | --- | --- | --- | --- | --- | --- | --- | --- | --- | --- | --- | --- | --- | --- | --- | --- | --- | --- | --- | --- | --- | --- | --- | --- | --- | --- | --- | --- | --- | --- | --- | --- | --- | --- | --- | --- | --- | --- | --- | --- | --- | --- | --- | --- | --- | --- | --- | --- | --- | --- | --- | --- | --- | --- | --- | --- | --- | --- | --- | --- | --- | --- | --- | --- | --- | --- | --- | --- | --- | --- | --- | --- | --- | --- | --- | --- | --- | --- | --- | --- |
