# Supplementary material for: Perceived coercion in psychiatric inpatients: a validation study of the Romanian-language version of the Admission Experience Survey
Source: Psychiatr Psychol Law. 2024 Sep 8;33(1):22–31. doi: 10.1080/13218719.2024.2372767 (PMC12857718; doi:10.1080/13218719.2024.2372767)
Supplement: Supplemental Material Romanian AES [file TPPL_A_2372767_SM6258.docx]

| **Chestionarul Admission Experience Survey (AES)**  "Vă voi citi o serie de afirmații privind venirea dumneavoastră la spital. Vă rog să răspundeți la fiecare afirmație cu „AEVĂRAT” sau „FALS”. Încercați să răspundeți cât mai exact la fiecare afirmație, oricât de similară ar părea cu altele.   \|  \| **Adevărat** \| **Fals** \| **Nu știu** \| \| --- \| --- \| --- \| --- \| \| **1.** Simt că am fost liber să fac cum am vrut în privința venirii la spital. \| **[ ]** \| **[ ]** \| **[ ]** \| \| **2.**Unele persoane au încercat să mă forțeze să vin la spital. \| **[ ]** \| **[ ]** \| **[ ]** \| \| **3.**Am avut ocazia să spun dacă vreau sau nu să vin la spital. \| **[ ]** \| **[ ]** \| **[ ]** \| \| **4.**A fost alegerea mea să vin la spital. \| **[ ]** \| **[ ]** \| **[ ]** \| \| **5.** Am putut spune ce am vrut legat de venirea la spital. \| **[ ]** \| **[ ]** \| **[ ]** \| \| **6.**Unele persoane m-au amenințat pentru a mă convinge să vin la spital. \| **[ ]** \| **[ ]** \| **[ ]** \| \| **7.** A fost ideea mea să vin la spital. \| **[ ]** \| **[ ]** \| **[ ]** \| \| **8.** Cineva a încercat să mă forțeze fizic să vin la spital. \| **[ ]** \| **[ ]** \| **[ ]** \| \| **9.** Nimeni nu părea interesat dacă eu eram de acord să merg la spital. \| **[ ]** \| **[ ]** \| **[ ]** \| \| **10.** Am fost amenințat că voi fi internat împotriva voinței mele. \| **[ ]** \| **[ ]** \| **[ ]** \| \| **11.** Mi s-a spus că voi fi obligat să vin la spital. \| **[ ]** \| **[ ]** \| **[ ]** \| \| **12.** Nimeni nu a încercat să mă forțeze să vin la spital. \| **[ ]** \| **[ ]** \| **[ ]** \| \| **13.** Părerea mea asupra venitului la spital nu a contat. \| **[ ]** \| **[ ]** \| **[ ]** \| \| **14.** Am putut controla în mare măsură dacă merg sau nu la spital. \| **[ ]** \| **[ ]** \| **[ ]** \| \| **15.**Eu am avut mai multă influență decât oricine altcineva asupra hotărârii de a veni la spital. \| **[ ]** \| **[ ]** \| **[ ]** \| \| **16.** Cum v-a făcut să vă simțiți faptul că ați fost internat în spital? \|  \|  \|  \| \| **a.** Furios \| **[ ]** \| **[ ]** \| **[ ]** \| \| **b.**Trist \| **[ ]** \| **[ ]** \| **[ ]** \| \| **c.** Mulțumit \| **[ ]** \| **[ ]** \| **[ ]** \| \| **d.**Ușurat \| **[ ]** \| **[ ]** \| **[ ]** \| \| **e.**Confuz \| **[ ]** \| **[ ]** \| **[ ]** \| \| **f.**Speriat \| **[ ]** \| **[ ]** \| **[ ]** \|   **Subscalele MacArthur Admission Experience Survey (AES)**  **I. Coerciție percepută**  **Pentru următoarele afirmații, fiecare răspuns „Adevărat” = 0 și fiecare „Fals” = 1 : 1,4,7,14,15**  Total =  **II. Presiuni negative**  **Pentru următoarele afirmații, fiecare răspuns „Adevărat” = 0 și fiecare „Fals” = 1. : 2,6,8,10,11**  **Pentru afirmația nr. 12, „Adevărat” = 1 și fiecare „Fals” = 0.**  Total =  **III. „Vocea” pacientului**  **Pentru afirmațiile 3 și 5, fiecare răspuns „Adevărat” = 0 și fiecare „Fals” = 1.**  **Pentru afirmația nr. 13, „Adevărat” = 1 și fiecare „Fals” = 0.**  Total =  Acest instrument este o traducere a scalei elaborate pe baza  Gardner, W., Hoge, S., Bennett, N., Roth, L., Lidz, C., Monahan, J., and Mulvey, E. (1993). Two scales for measuring patients' performance perceptions of coercion during hospital admission. *Behavioral Sciences and the Law, 20,* 307-321. |
| --- | --- | --- | --- | --- | --- | --- | --- | --- | --- | --- | --- | --- | --- | --- | --- | --- | --- | --- | --- | --- | --- | --- | --- | --- | --- | --- | --- | --- | --- | --- | --- | --- | --- | --- | --- | --- | --- | --- | --- | --- | --- | --- | --- | --- | --- | --- | --- | --- | --- | --- | --- | --- | --- | --- | --- | --- | --- | --- | --- | --- | --- | --- | --- | --- | --- | --- | --- | --- | --- | --- | --- | --- | --- | --- | --- | --- | --- | --- | --- | --- | --- | --- | --- | --- | --- | --- | --- | --- | --- | --- | --- | --- |
